# The Attitude of Medical Students Toward Complementary Medicine: Results of a Cross-Sectional Study

**DOI:** 10.1089/acm.2021.0181

**Published:** 2021-12-03

**Authors:** Gabriele Rotter, Lea Jerzynski, Maximilian Hinse, Sylvia Binting, Benno Brinkhaus

**Affiliations:** Charité—Universitätsmedizin Berlin, Corporate Member of Freie Universität Berlin and Humboldt-Universität zu Berlin, Institute of Epidemiology and Health Economics, Berlin, Germany.

**Keywords:** complementary medicine, integrative medicine, medical students, cross-sectional study, prevalence

## Abstract

***Introduction:*** Complementary medicine (CM) is often used by patients and offered by physicians. The attitude of medical students toward CM in Germany has been given little research attention. The aim was to assess the attitude of medical students toward CM in general and their opinion about the importance of CM university research and teaching.

***Methods:*** An exploratory cross-sectional study among medical students at the Charité—Universitätsmedizin Berlin was performed at the beginning of the summer term 2019 using an online survey. The attitude toward CM was assessed by the Complementary and Alternative Medicine Health Belief Questionnaire (CHBQ, range 10–70, neutral at 40; a higher score indicates a more positive attitude toward CM). Furthermore, students rated their own CM use and the perceived importance of CM university research and teaching (range 1–7; a higher score indicates more agreement). The study was approved by the Charité Ethics Committee (institutional review board).

***Results:*** Out of 1256 contacted students, 349 (27.8%) students (mean age 23.7 ± 4.3 years, 69.0% female) participated. The attitude toward CM based on the CHBQ was rather neutral (mean 44.2 ± 10.7) and more positive among females than males (mean 46.1 ± 10.7 vs. 40.6 ± 9.5, *p* < 0.001). Medical students favored CM university research (mean 5.4 ± 1.5) and mostly did not agree that CM is currently taught sufficiently at the university (mean 3.4 ± 1.7). The lifetime prevalence of student's own CM use was 48.4% of respondents (79.1% females).

***Conclusion:*** Although medical students, in this sample with a high percentage of females, reported a rather neutral attitude toward CM, the authors' findings indicate that medical students promoted research and teaching in CM. Further multicenter cross-sectional studies in German and European medical universities should be undertaken to explore students' attitudes and wishes regarding the integration of CM in university teaching, research, and patient care.

## Introduction

Complementary medicine (CM) covers a heterogeneous group of diagnostic and therapeutic procedures^1^ that are not part of but can be used in combination with conventional medicine.^2^ European CM use ranges from 0.3% to 86%.^3,4^ In Germany, more than 60%–85% of physicians offer CM or refer to it.^5,6^ In the United States of America, the Complementary and Alternative Medicine Health Belief Questionnaire (CHBQ)^7^ has been validated to survey attitudes toward CM among medical students. Since then, the CHBQ has been used internationally in students of various professions.^7–12^ However, its use in Germany has not been investigated and published, to the best of the authors' knowledge.

Although the topic is still highly polarized, CM is increasingly integrated into university teaching. A combination of CM teaching and practical experience might contribute to the development of a holistic patient-oriented attitude among medical students.^13^ The ninth revision of the German Medical Licensing Regulations in 2003 facilitates teaching in naturopathy as part of the CM in medical universities. At Charité—Universitätsmedizin Berlin, the New Revised Medical Curriculum (“Modellstudiengang 2.0,” 2015) follows a competence-based teaching approach.^14^ Medical students attend the university for 6 years, and the first 5 years contain theoretical and practical studies. The sixth year is a “practical year” (final year rotation). Medical students' attitudes toward CM and their CM use have received little research attention in Germany.

The study aim was to assess the attitude of medical students in Berlin toward CM in general and their opinion about the importance of CM university research and teaching.

## Methods

### Study design and setting

An exploratory cross-sectional study among medical students in the first and second semesters (first year) and the 9th and 10th semesters (fifth year) at the Charité—Universitätsmedizin Berlin was performed online at the beginning of the summer term (for <6 weeks) by the Institute for Social Medicine, Epidemiology, and Health Economics. The study followed the standards of the Declaration of Helsinki^15^ and the International Council for Harmonisation of Technical Requirements for Pharmaceuticals for Human Use (ICH)-good clinical practice (GCP) guidelines,^16^ and was approved by the Charité Ethics Committee (approval number EA1/033/19). All participants were informed about the study and data protection by an online text and gave informed consent before inclusion in the study.

### Participants

To recruit participants, in-house e-mail distribution, closed Facebook groups and in-house posters were used. As an incentive, 10 book vouchers, worth 30€ each, were drawn among the participants. For the distribution of the prize, students could voluntarily provide an e-mail address, which was collected separately and not assignable to other data. Only medical students of human medicine (hereafter referred to as “students”) enrolled at Charité—Universitätsmedizin Berlin were included. Further inclusion criteria were being 18–40 years old and being enrolled in semesters 1, 2, 9, or 10 at the time of the survey. An internet-capable device (e.g., smartphone, computer, and tablet) was a prerequisite for participation. The exclusion criteria were being outside the specified ages and semesters.

### Outcome measurement and data collection

The collection of sociodemographic data was limited to age, semester attended, and gender.

The students' attitude toward CM was assessed by the 10-item CHBQ.^7^ It applies 7-point scales (1 = absolutely disagree, 7 = absolutely agree, total score range 10–70, neutral 40 points). A higher score indicates a more positive attitude toward CM. No validated German translation could be found. Therefore, the CHBQ was translated into German and back-translated into English in agreement among four researchers: three native German speakers and one native English speaker.

To obtain indications on the students' opinion on CM integration into university research and teaching, three statements were presented: “CM should be scientifically researched at the university”; “CM should be integrated into university teaching”; and “CM is satisfactorily taught at the university.” The students expressed their opinion using a 7-point scale (1 = “I do not agree at all,” 7 = “I totally agree”). In addition, the students were asked to indicate whether they had studied CM themselves in addition to the obligatory part (yes, no), for example, whether they had read specialized journals or taken an elective/additional course. Regarding CM in their future career, the students answered the question “As a physician, I could imagine offering CM to my patients.” For a selection of CM methods (acupuncture, osteopathy, homeopathy, and naturopathy), the students rated on a 7-point scale (1 = “I do not agree at all,” 7 = “I totally agree”) the extent to which they could imagine offering the respective method once they become physicians. The values on the 7-point scales were interpreted as follows: 1–3 points indicate rejection, 5–7 points indicate agreement, and 4 points indicate a neutral attitude.

To obtain data on the students' own CM use during their lifetime and the last 12 months, a selection of 16 different CM methods (acupuncture, acupressure, Tai Chi/*qigong*, Chinese medicine, osteopathy, chiropractic, manual therapy, herbal remedies, food supplements, diet, hypnosis, yoga, meditation, homeopathy, Ayurveda, anthroposophic medicine, and “other”) was listed. Twelve of these were also listed in the CHBQ validation study.^7^ The authors added *acupressure, manual therapy, dietary changes,* and *anthroposophic medicine* because they are often used in Germany.

The data were collected anonymously by using an online survey (SoSci Survey).^17^ A definition of CM was not provided to the students in the introduction to the questionnaire.

### Statistical analysis

As this is an exploratory cross-sectional study, a sample size calculation was not performed. The authors aimed to include ∼300 students because this was the approximate count of participants enrolled in the CHBQ validation study.^7^ The evaluation of the data was quantitative, descriptive, and explorative. The data were evaluated descriptively using mean values, standard deviation, and absolute and relative frequencies. Mean values and frequencies were tested for group differences using the *t* test for independent samples (CHBQ, CM integration into university research and teaching, CM in future career) or the chi-square test (CM use, self-studies in CM), according to the scales. The results are reported as group means with 95% confidence intervals (95% CIs) and the *p*-value for the group comparison. The significance level was established as 5% (*p* < 0.05). All tests were two sided. All *p*-values were considered explorative without adjustment for multiple testing. Cohen's *d* was calculated for CM integration into university teaching and research. In addition, stratified analyses were performed using the variables gender and study year (first year vs. fifth year). The CHBQ contains three statements (numbers 6–8) that are reverse scored and required recoding for evaluation. Regarding missing data, no imputation was done. Statistical analyses were performed by IBM SPSS for Windows Version 25,^18^ R version 4.0.0,^19^ and RStudio version 1.2.5042.^20^

## Results

### Participants

**FIG. 1.**
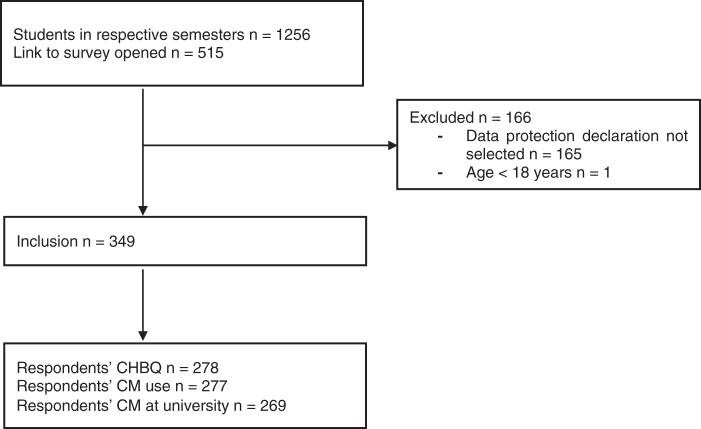
Recruitment and respondents. CHBQ, Complementary and Alternative Medicine Health Belief Questionnaire; CM, complementary medicine.

Between April 8 and May 14, 2019, out of 1256 approached students (60.9% female), 349 (27.8%) students (mean age 23.7 ± 4.3 years, 69.0% female) were included (Fig. 1, Table 1). Among them, 313 students reported their semester attended: 149 attended the first year, and 164 studied in the fifth year.

**Table 1.** Characteristics of the Study Population

	*n*	Mean ± SD,* n *(%)
Age	297	23.7 ± 4.3
Gender	316	
Female		218 (69.0)
Male		94 (29.7)
Not specified		4 (1.3)
Semester	313	
First (first year)		65 (20.8)
Second (first year)		84 (26.8)
Ninth (fifth year)		74 (23.6)
10th (fifth year)		90 (28.8)

SD, standard deviation.

### Students' attitude toward CM based on the CHBQ

The CHBQ was completed by 278 students (191 females and 83 males). The attitude toward CM based on the CHBQ was rather neutral (mean 44.2 ± 10.7, Table 2) and more positive among females than males (mean 46.1 ± 10.7 vs. 40.6 ± 9.5, *p* < 0.001). There were no differences between the first and fifth years.

**Table 2.** Complementary and Alternative Medicine Health Belief Questionnaire Score Results, Opinions of Students Toward Complementary Medicine Regarding Integration into University Research and Teaching, and Offerings in Their Future Careers

	*n*	Total Mean ± SD* n *(%)	Female Mean ± SD* n *(%)	Male Mean ± SD* n *(%)	Female vs. male* p*-value	First year Mean ± SD* n *(%)	Fifth year Mean ± SD* n *(%)	First vs. fifth year *p*
CHBQ score^a^	278	44.2 ± 10.7	46.1 ± 10.7	40.6 ± 9.5	<0.001	45.1 ± 9.7	43.4 ± 11.6	0.140
CM in university (1–7)^b^	269							
CM should be scientifically researched at the university		5.4 ± 1.5	5.4 ± 1.5	5.2 ± 1.6	0.189	5.4 ± 1.3	5.3 ± 1.7	0.780
CM should be integrated into university teaching		4.4 ± 1.9	4.8 ± 1.8	3.5 ± 1.9	<0.001	4.5 ± 1.8	4.3 ± 2.0	0.420
CM is satisfactorily taught at the university		3.4 ± 1.7	3.2 ± 1.7	3.8 ± 1.6	<0.050	3.2 ± 1.3	3.6 ± 1.9	0.108
CM self-studies	269	101 (37.5)	75 (39.9)	24 (31.2)	0.230	32 (25.0)	69 (48.9)	<0.010
CM in future career (1–7)^b^								
Acupuncture	264	5.0 ± 1.8	5.1 ± 1.8	4.7 ± 1.8	0.112	5.2 ± 1.6	4.8 ± 2.0	0.080
Osteopathy	259	4.2 ± 2.0	4.4 ± 2.0	3.9 ± 1.8	<0.050	4.6 ± 1.8	3.9 ± 2.1	<0.010
Homeopathy	261	3.2 ± 2.1	3.6 ± 2.1	2.4 ± 1.8	<0.001	3.8 ± 2.1	2.7 ± 1.9	<0.001
Naturopathy	263	4.0 ± 2.3	4.7 ± 1.9	3.7 ± 1.9	<0.001	4.5 ± 1.9	4.3 ± 2.0	0.573

^a^ (Range 10–70, neutral 40 points) a higher score indicates a stronger positive attitude toward CM.

^b^ (Range 1–7, 1 = I do not agree at all, 7 = I totally agree, 4 = neutral).

CHBQ, Complementary and Alternative Medicine Health Belief Questionnaire; CM, complementary medicine; SD, standard deviation, % are valid percent.

### Students' opinion on CM integration into university research and teaching

Students favored CM integration into university research (mean 5.4, standard deviation [SD] ±1.5, Table 2). The majority (76.6%) agreed. There was no difference in gender regarding the opinion on CM integration into university research (female vs. male mean difference = 0.28, 95% CI [−0.70 to 0.14], *p* = 0.189, Cohen's *d* = −0.23). In contrast, CM integration into university teaching was rated more neutral (mean 4.4, SD ±1.9), with agreement of more than half (54.3%) of the respondents. Females supported CM integration more than males (mean difference = 1.30, 95% CI [−1.79 to −0.81], *p* < 0.001, Cohen's *d* = −0.92). The students perceived the current CM university teaching as insufficient (mean 3.4, SD ±1.7). Only 22.9% of the students rated the teaching as sufficient. Female students considered the university teaching on CM to be less sufficient than male students did (mean difference = −0.54, 95% CI [0.12–1.00], *p* < 0.050, Cohen's *d* = 0.43). More than one-third (37.5%) had engaged in self-study of CM, with 25.0% of first-year students and 48.9% of fifth-year students (*p* < 0.001).

### CM in future career

The majority of the responding students could imagine possibly offering CM as doctors to their patients. For acupuncture, 68.8% of students agreed, with a mean of 5.0 (SD ±1.8). Approximately half of the students could imagine offering naturopathy (51.3% agreed, mean 4.0, SD ±2.3) or osteopathy (46.7% agreed, mean 4.2, SD ±2.0), and approximately one-third (29.5% agreed, mean 3.2, SD ±2.1) intended to offer homeopathy later in their career.

### Students' own CM use

During their lifetime, 134 (48.4%) of the responding students used CM, and 106 (79.1%) were females. Lifetime CM use of responding students was comparable between the first year and the fifth year (46.2% vs. 50.3%, *p* = 0.492). Furthermore, 132 students specified the CM method used; the following percentages of CM use in this section refer to these 132 respondents: During the students' lifetime, herbal remedies (72.0%), homeopathy (56.8%), and yoga (57.6%) were used most often (Table 3). Approximately two-thirds of the female CM users had done yoga (66.7%) or used homeopathy (62.9%). Male CM users applied herbal remedies (57.7%) and acupuncture (38.5%) most frequently. Almost half (46.2%) of the respondents had used five or more CM methods. During the last 12 months, 72% of CM users used at least one CM method, 57.6% used at least two, and 22.0% used at least five. During the last 12 months, herbal remedies, yoga, and dietary supplements were most often used in the whole sample and by students in the fifth year. Students in the first year used herbal remedies and homeopathy the most. Only yoga was used significantly less by first-year students than by fifth-year students (15 (25.0%) vs. 32 (44.4%), *p* = 0.078).

**Table 3.** Students' Own Complementary Medicine Use During Their Lifetime and the Last 12 Months

	Total* n* = 132* n *(%)	Female* n* = 105* n *(%)	Male* n* = 26* n *(%)	*p*-value male vs. female	First year* n* = 149* n *(%)	Fifth year* n* = 164* n *(%)	*p*-value first year vs. fifth year
Acupuncture during lifetime	58 (43.9)	48 (45.7)	10 (38.5)	0.830	21 (35.0)	37 (51.4)	0.057
Last 12 months	20 (15.2)	15 (14.3)	5 (19.2)	0.692	9 (15.0)	11 (15.3)	1.000
Acupressure (lifetime)	25 (18.9)	21 (20.0)	4 (15.4)	0.831	6 (10.0)	19 (26.4)	0.021
Last 12 months	16 (12.1)	14 (13.3)	2 (7.7)	0.707	5 (8.3)	11 (15.3)	0.247
Tai Chi/*qigong* (lifetime)	16 (12.1)	15 (14.3)	1 (3.8)	0.289	7 (11.7)	9 (12.5)	0.962
Last 12 months	4 (3.0)	3 (2.9)	1 (3.8)	1.000	1 (1.7)	3 (4.2)	0.793
Chinese medicine (lifetime)	25 (18.9)	17 (16.2)	8 (30.8)	0.178	6 (10.0)	19 (26.4)	0.043
Last 12 months	6 (4.5)	4 (3.8)	2 (7.7)	0.770	2 (3.3)	4 (5.6)	0.910
Osteopathy (lifetime)	47 (35.6)	39 (37.1)	8 (30.8)	0.627	18 (30.0)	29 (40.3)	0.425
Last 12 months	22 (16.7)	18 (17.1)	4 (15.4)	1.000	9 (15.0)	13 (18.1)	0.940
Chiropractic (lifetime)	25 (18.9)	21 (20.0)	4 (15.4)	0.746	9 (15.0)	16 (22.2)	0.505
Last 12 months	4 (3.0)	2 (1.9)	2 (7.7)	0.386	2 (3.3)	2 (2.8)	1.000
Manual therapy (lifetime)	45 (34.1)	37 (35.2)	8 (30.8)	0.910	16 (26.7)	29 (40.3)	0.095
Last 12 months	20 (15.2)	16 (15.2)	4 (15.4)	1.000	6 (10.0)	14 (19.4)	0.153
Herbal remedies (lifetime)	95 (72.0)	79 (75.2)	15 (57.7)	0.236	43 (71.7)	52 (72.2)	1.000
Last 12 months	56 (42.4)	50 (47.6)	6 (23.1)	0.053	23 (38.3)	33 (45.8)	0.778
Food supplements (lifetime)	63 (47.7)	55 (52.4)	7 (26.9)	0.054	23 (38.3)	40 (55.6)	0.149
Last 12 months	42 (31.8)	41 (39.0)	0 (0.0)	<0.001	17 (28.3)	25 (34.7)	0.808
Diet (lifetime)	55 (41.7)	48 (45.7)	6 (23.1)	0.113	21 (35.0)	34 (47.2)	0.136
Last 12 months	33 (25.0)	29 (27.6)	3 (11.5)	0.175	12 (20.0)	21 (29.2)	0.258
Hypnosis (lifetime)	13 (9.8)	9 (8.6)	4 (15.4)	0.423	5 (8.3)	8 (11.1)	1.000
Last 12 months	5 (3.8)	3 (2.9)	2 (7.7)	0.386	2 (3.3)	3 (4.2)	1.000
Yoga (lifetime)	76 (57.6)	70 (66.7)	6 (23.1)	<0.001	26 (43.3)	50 (69.4)	0.015
Last 12 months	47 (35.6)	44 (41.9)	3 (11.5)	0.007	15 (25.0)	32 (44.4)	0.078
Meditation (lifetime)	57 (43.2)	49 (46.7)	8 (30.8)	0.348	21 (35.0)	36 (50.0)	0.111
Last 12 months	33 (25.0)	28 (26.7)	5 (19.2)	0.608	10 (16.7)	23 (31.9)	0.142
Homeopathy (lifetime)	75 (56.8)	66 (62.9)	9 (34.6)	0.041	39 (65.0)	36 (50.0)	0.057
Last 12 months	30 (22.7)	27 (25.7)	3 (11.5)	0.175	18 (30.0)	12 (16.7)	0.067
Ayurveda (lifetime)	13 (9.8)	12 (11.4)	1 (3.8)	0.563	5 (8.3)	8 (11.1)	0.834
Last 12 months	5 (3.8)	5 (4.8)	0 (0.0)	0.693	1 (1.7)	4 (5.6)	0.793
Anthroposophic medicine (lifetime)	22 (16.7)	20 (19.0)	2 (7.7)	0.399	7 (11.7)	15 (20.8)	0.355
Last 12 months	9 (6.8)	8 (7.6)	1 (3.8)	0.909	4 (6.7)	5 (6.9)	1.000
Other (lifetime)	13 (9.8)	10 (9.5)	3 (11.5)	1.000	6 (10.0)	7 (9.7)	0.834
Last 12 months	3 (2.3)	3 (2.9)	0 (0.0)	0.873	2 (3.3)	1 (1.4)	0.829

## Discussion

This exploratory cross-sectional study provides an overview of the CM attitudes of ∼300 medical students, with a high percentage of females, in Germany in the first and fifth study years. The attitudes toward CM measured by the CHBQ were rather neutral. The students favored CM university research. The students promoted CM integration into university teaching but rated their current CM university teaching as rather insufficient. More than one-third had studied CM on their own. In their future career, most responding students were able to imagine offering a CM method to patients, especially acupuncture. More than one-third had used CM themselves during their lifetime.

This study is, to the authors' knowledge, the first German study using the CHBQ with German medical students, and the authors first provide a German version of the internationally used CHBQ.^7–12^ The study further gives a comprehensive overview of the attitude of German medical students toward CM, their opinion on CM university research and teaching, their perception of later CM recommendation in medical practice, and their lifetime and 12-month prevalence of CM use. The rather equal distribution of the study semesters per year, the short study time, and the monocenter design provided a quite homogenous study population, which augments the internal validity. Although this was not a cohort study, the use of students from the first and fifth years provides at least some impression of the possible development of CM attitudes during medical studies in Germany.

The main limitation is the overall response rate of approximately one-fourth of contacted students. This could have led to an overestimation of the reported results. Students more interested in CM might have been more likely to participate. However, German studies in the general population and a young population of athletes have reported higher CM use^21–23^ than the authors found in their study. The low response rate in this study might be due to the planned short period of study to obtain results at the beginning of a semester, but also to low interest in the subject of CM by students, in addition to the fact that the recruitment occurred through media rather than directly in classes, as done in another study.^7^ A further limitation is that not all parts of the survey were answered by all participants. However, the survey was completely filled out by most respondents, and the data were reliable. Although the monocentric study design augments the internal validity of the study, no general conclusions for Germany are possible. Moreover, different teaching curricula are applied in Germany. Another limitation is that the authors did not provide a definition of CM to the participants. This diminishes the internal validity of the results regarding attitudes toward CM. Another limitation is the cross-sectional nature, because the retrospective evaluation of CM use during the lifetime and the last 12 months entail a risk of recall bias, which could have led to an underestimation of this lifetime and 12-month prevalence.

In this population, the authors rated the medical students' attitude toward CM to be neutral, with a CHBQ mean of 44.2 ± 10.7. In contrast, in Germany, more than 60%–85% of physicians offer CM or refer to it.^5,6^ Medical students at Charité—Universitätsmedizin Berlin receive teaching in CM and also receive teaching in research methodology. In the authors' opinion, the medical students' more neutral attitude toward CM and demand for CM research reflect the critical awareness of the students toward CM, which may be driven by the critical attitude toward CM by the Charité professors and the teaching stuff. However, other studies found positive attitudes with slightly more elevated CHBQ values. In 272 U.S. medical students (response rate of 96.5%), a positive attitude was found, with a CHBQ mean of 47.8 ± 8.1.^7^ A recent cross-sectional study in China^9^ included 2292 students at medical and nonmedical universities and reported a mostly positive attitude, with a CHBQ mean 48.9 ± 8.6. Further studies on medical students reported equally positive CM attitudes of first- and second-year students, with more negative attitudes toward CM in third-year students^24^; a positive CM attitude, with 92% believing that CM includes ideas and methods from which conventional medicine can benefit^25^; and varying beliefs about CM.^26^ In addition, attitudes were evaluated with the CHBQ in students from other faculties. In the Czech Republic, a rather positive CM attitude based on the CHBQ was reported for pharmacy students.^11^ In Serbia, various groups from the health care system, including medical, dental, and pharmacy students, reported positive CM attitudes based on the CHBQ.^12^ In Australia, CHBQ scores for nursing and chiropractic students indicated relatively positive CM attitudes.^10^

Female respondents in this study reported a more positive CM attitude than males. This is in line with the literature and it is known that females prefer CM more than males.^7,22,27^

CM integration into university teaching in Berlin was supported,^26^ as students reported having poor CM knowledge across the study program^25^ or that students earlier in their program had better CM knowledge.^26^ Furthermore, apart from an optional course in teaching CM in the third semester, in students' working group focusing on CM during the whole study period, it is late in the 10th semester that students learn the principles of CM. However, agreement about CM integration into university teaching in this study (54.3%) was rather low compared with that found in other surveys reporting a rate of more than 80%.^9,25^ Moreover, a narrative review of 21 articles pointed out that medical students regard CM as highly relevant in medical education and later patient care but appear to have lower levels of knowledge of CM than other providers.^28^ Because of the rise of CM use in the population, further inclusion of CM into medical curricula needs consideration.^28^ This should be accompanied by sufficient university CM research. The students in this study strongly supported CM integration into university research. In the authors' opinion, this reflects the critical awareness of the students toward CM. Regarding CM integration into their future career as doctors, this study respondents could imagine offering acupuncture (two-thirds of students), naturopathy (half), osteopathy (half), and homeopathy (one-third). In a study among general practitioners in Germany (response rate 46%, random sample), 85% of respondents reported using CM at least once a week, most often herbal medicines, vitamins/supplements, and homeopathic remedies.^6^ A qualitative study with 13 young German general practitioners reported that they were generally open to CM, especially to herbal medicine.^29^

Internationally, more than 91% of medical students were positive about integrating CM into their future career.^25^ Palestine medical students already frequently recommend CM, mostly massage therapy, herbal medicine, yoga, and chiropractic.^26^ Compared with international studies in medical students, which reported CM use at 73.5% (272 students, United States)^7^ and 61.8% (1448 students, China),^9^ CM use (48.4%) by medical students in Berlin was lower. Furthermore, CM use in Germany was reported to be higher, with more than 60% in the general population^21,22^ and in a young population of high-performing athletes.^23^

Future studies should focus on prospectively evaluating students' developing attitudes toward CM, their opinions on CM university research and teaching during their studies, and their CM use. Therefore, future studies on this topic should use not only cross-sectional but also prospective multicenter cohort design. For this, the authors advise considering “in-class” distributions and filling out the assessment tools to obtain a high response rate. Deeper knowledge of medical students' CM attitudes could aid in further adapting university curricula to students' needs to develop a comprehensive, holistic, and patient-oriented attitude for their later careers.

## Conclusion

Although medical students, in this sample with a high percentage of females, reported a rather neutral attitude toward CM, the authors' findings indicate that medical students promote research and teaching in CM. Further multicenter cross-sectional studies in German and European medical universities should be undertaken to explore students' attitudes and wishes regarding the integration of CM in university teaching, research, and patient care.
